# Classification of Diapause Status by Color Phenotype in *Lygus hesperus*

**DOI:** 10.1673/031.012.13601

**Published:** 2012-11-26

**Authors:** Colin S. Brent

**Affiliations:** USDA-ARS, Arid Land Agricultural Research Center, 21881 N. Cardon Lane, Maricopa, AZ 85138

**Keywords:** overwintering, polyphenism, western tarnished plant bug

## Abstract

Recent studies on adult diapause in the western tarnished plant bug, *Lygus hesperus* Knight (Heteroptera: Miridae), have highlighted the need to identify a reliable external marker for the internal changes that differentiate a normal animal from one that is overwintering. To test the efficacy of a color based discrimination system, *L. hesperus* of both genders were reared from eggs through day 10 of adulthood under a 10 hour photophase at a constant temperature. They were separated into three color groups (yellow, pale green, dark green), then dissected for diapause categorization based on internal development. Most yellow individuals were in diapause, dark green individuals were not, and pale green ones were mixed. A group of 25 assessors, naïve with regard to *L. hesperus* development, were then asked to use a simplified color criteria (yellow = diapause, green = non-diapause) to estimate the status of a mixture of diapausing and non-diapausing adults of both genders aged two to seven days post-eclosion. After dissection to verify diapause status, assessor accuracy was found to be ineffective for assessing adults of both sexes younger than four days because color differences, which increased with age, were subtle or non-existent at this stage. For four to seven-day-old bugs, 84% of females and 67% of males were correctly categorized, on average. Incorrect assessments in all but the youngest males over-identified diapause, but for females there was no trend in miscategorizations. Overall, the results indicate that diapause status can be adequately discriminated by color assessment, and with greatest accuracy when sampling older females. However, factors other than photoperiod appear to also influence coloration.

## Introduction

The western tarnished plant bug, *Lygus hesperus* Knight (Heteroptera: Miridae), is a pest of numerous fiber, fruit, seed, and vegetable crops (Jackson et al. 1995), and is found throughout the southwestern United States. It is a multivoltine species, and during the winter months it produces a generation of overwintering adults (Beards and Strong 1966). After exposure to a token stimulus of short photophases during their sensitive nymphal stage, these individuals change their developmental trajectory to enter diapause as adults (Beards and Strong 1966; Leigh 1966; Spurgeon and Brent 2010). Diapause is a genetically controlled dormancy that in many insects reduces feeding and reproductive development to enhance survival during extended periods without access to food (Tauber et al. 1986; Denlinger et al. 2005). The diapause response of *L. hesperus* includes hypertrophy of the fat body, and delayed maturation of the female ovaries and male accessory glands (Beards and Strong 1966; Strong et al. 1970; Spurgeon and Brent 2009). There are other accompanying behavioral and physiological changes, many of which are only recently being explored. It is thought that such changes might leave insects more susceptible to some ecologically or culturally based management tactics (McGuire et al. 2006). However, development of new management tactics to target overwintering *L. hesperus* populations will require a means to accurately and rapidly assess the diapause status of field populations without having to examine their internal development.

One possible external marker that may serve this purpose is a color diphenism that has been noted in *L. hesperus* reared under short day photoperiods (Beards and Strong 1966; Strong et al. 1970). Seasonal color differences, along with other diapause-mediated polyphenisms, occur in a number of insects (Tauber et al. 1986), including other Heteroptera (see below). For both female and male *L. hesperus*, non-diapausers normally develop an abdomen that is bright or dark green as they mature, while diapausers are often pale green or light yellow. While this general pattern has been observed, the strength of the association has not been determined. Additionally, it is not known whether this color difference results from the delayed onset of normal color change in diapausers (e.g., Fasulati 1979), or from active pigmentation processes that produce divergent phenotypes (e.g., Niva and Takeda 2002). The color difference in *L. hesperus* appears related to the development of the fat body, which is usually aqua in non-diapausers and white in diapausers (Spurgeon and Brent 2010). However, color differences in the fat body were not consistently associated with reproductive diapause in this previous study. Although the relationship of diapause status to external body color was never directly assessed for *L. hesperus*, this inconsistency raises the possibility that a change in body color may be a response to seasonal stimuli that is decoupled from the state of the reproductive organs, as has been noted in other insects (Tauber et al. 1986; Danks 1987; Seymour and Bowman 1994; Mourão and Panizzi 2002). Among the Heteroptera studied, seasonal color changes in diapausing individuals were sometimes strongly linked to reproductive activity (Musolin and Namuta 2003, 2004), but in other cases, there was evidence of additional modulation by factors such as age (Niva and Takeda 2002; Chocorosqui and Panizzi 2003), ambient temperature (Wilborn and Ellington 1984; Kobayashi and Numata 1995; Kotaki 1998), gender, genetics (Niva and Takeda 2002), and social interactions (Harris et al. 1984).

The objective of this study was to determine how strongly abdomen color was coupled to diapause status in *L. hesperus*, and to determine if color differences were influenced by age and gender. With the goal of developing an effective tool for categorizing insects under field conditions, the study also measured the accuracy with which minimally trained human observers could use simple color criteria to categorize mixed groups of *L. hesperus*. The results highlight both the weaknesses and strengths of using color discrimination for diapause status, and begin to elucidate some of the factors other than photoperiod that modulate seasonal color change.

## Materials and Methods

### Insects

The experiment was conducted at the United States Department of Agriculture, Agricultural Research Service laboratory in Maricopa, AZ, USA, where an *L. hesperus* colony has been under continuous culture for more than 10 years, with periodic introductions of field collected adults to maintain vigor. The stock insects were held at 27.5–29.0° C under a 14-hour photophase, and provisioned with artificial diet (Debolt 1982) packaged in Parafilm M (Pechiney Plastic Packaging, Chicago, IL) (Patana 1982). Eggs were obtained by placing oviposition packets, agarose gel packaged with Parafilm M, on rearing cages for 6–8 hours. The oviposition packets were placed in 1890 mL waxed chipboard cups (Huhtamaki, http://www.us.huhtamaki.com) held in an environmental chamber, and maintained at a 10:14 L:D photoperiod at 27.0 ± 1° C. Emerging nymphs were provided an *ad libitum* diet of green bean pods (*Phaseolus vulgaris* L.), and held in mixed-sex groups. Beans were replenished three times weekly, or more often if their quality deteriorated. The number of individuals in each container was kept below 150 to ensure normal development (Brent 2010a). Beginning when fifth instars were first observed, nymphs were monitored daily to detect adult eclosion. Adults emerging on the same day were separated by gender, based on the presence or absence of an ovipositor, and placed into separate rearing containers. These adults were reared under the same conditions as the nymphs.

### Association between abdomen color and diapause status

**Figure 1.**
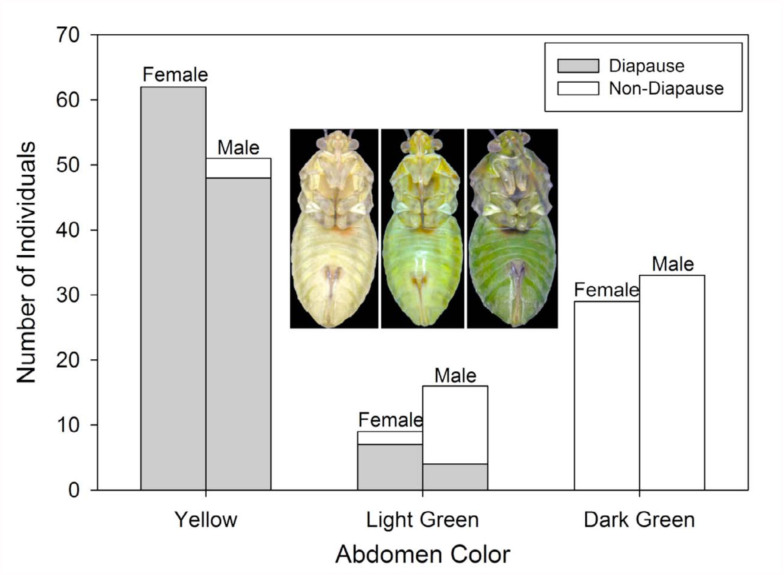
Number and relative color distribution of diapausing and non-diapausing female and male Lygus *hesperus* sampled 10 days after adult emergence. Representative color phenotypes from 10-day-old females are shown. All individuals were reared simultaneously under identical conditions. For each gender, 100 individuals were sampled. High quality figures are available online.

To test the association between abdomen color and diapause status, a group of 100 males and 100 females were reared to ten days post-eclosion. Each individual was then assigned to one of three color categories (yellow, light green, dark green; Figure 1). This was done solely by the author to ensure consistent categorization. Following color assessment, each adult was dissected in saline (0.7% NaCl (wt:vol)) to determine whether it was in diapause. Adult diapause status was characterized based on the condition of the fat body and reproductive organs (Spurgeon and Brent 2010; Brent and Spurgeon 2011). Relaxed diapause criteria, as defined in Brent and Spurgeon (2011), were used in the classification. To be designated as diapausing, individuals had to have a hypertrophied fat body. Additionally, females could not have mature eggs or follicular relics in their ovaries, and males had to have undeveloped lateral and underdeveloped medial accessory glands.

### Accuracy of color assessment in young *L. hesperus*

To determine the accuracy with which abdomen color could be used to assess diapause status in less mature *L. hesperus*, additional adults were reared under the same short day conditions. On each day for the two to seven days after adult eclosion, 25 randomly drawn individuals of each sex were flash-frozen in liquid nitrogen, then placed in a -80° C freezer for one hour prior to assessment. Rapid low temperature freezing helped to preserve the color of the insects so that all the animals collected from a single cohort could be assessed simultaneously. Because fewer than 60% of individuals reared under the specified conditions will enter diapause (Brent and Spurgeon 2011), each set of 25 contained a mixture of diapausers and non-diapausers. Each insect was placed into a clear container numbered for identification purposes.

The 12 groups (one of each sex for each of six days) of 25 insects were then presented to individuals asked to determine diapause status based on color alone. Assessors were initially naïve about the putative color differences between diapausers and non-diapausers, and most had limited experience with lygus bugs. They were told that the color of the ventral abdomen ranges from pale yellow to light green in diapausers, and from medium to dark green in non-diapausers. They were asked to ignore cuticular pigmentation on the male abdomen, and to focus on the bands of color that ran along the margins of the abdomen. The black pigmentation, which develops on the central region of the abdomen of male *L. hesperus*, changes with age, and does not appear to be dependent on diapause status (personal observation). No visual aids, such as magnifiers, were allowed, and all insects had to be assigned to one of the two status categories. Several individuals simultaneously assessed the insects, and each received the grouped insects in a randomized order to avoid learning associated changes in identification accuracy. Replicates of the experiment were run on four separate dates, each using *L. hesperus* from a different cohort and a different group of assessors. A total of 25 different assessors were used.

Pictures were taken of a subset of the diapausing and non-diapausing individuals under magnification (1.6x, using a Leica DFC425 camera attached to a Leica M165C microscope with an LED ring light). For each day and gender, 18–25 individuals were photographed. The brightness of the abdomen for females and males at each age was determined using the “luminosity” reading on Photoshop Elements v. 5.0.2 (Adobe Systems Inc., http://www.adobe.com). Readings were taken from four discreet 100 × 100 pixel squares located along the non-pigmented margins of the abdomen at the second and seventh sternites, and from one 1000 × 1000 pixel square that covered the majority of the abdomen (Figure 5).

As with the 10-day-old adults, the actual diapause status of these younger individuals was determined by dissection. A female was classed as diapausing if she exhibited no evidence of vitellogenesis, and had a well developed, if not necessarily hypertrophied, fat body. A male was classed as diapausing if he contained undeveloped lateral and medial accessory glands that were small and lacked colored contents. Any evidence of activity by these organs was cause for a non-diapause classification. Stringent criteria, as defined in Brent and Spurgeon (2011), were used because males were housed separately from females, preventing mating and the depletion of the accessory gland contents. Because fat body development increased with age in all adults (Spurgeon and Brent 2010), greater hypertrophy was required for older individuals to be classed as diapausing.

### Statistical Analyses

The association between gender and diapause status was examined using a chi-square test. The association between abdomen color and diapause status for 10-day-old adults was examined in a contingency table separately for each gender using the two-sided Fisher Exact test. The overall association between color and diapause was tested with the Cochran-Mantel-Haenszel nonzero correlation statistic using the Yates correction for continuity (*χ^2^ CMH*, Sokal and Rohlf 1995). Because of a large increase in the correct identification of diapause status in individuals older than four days, chi-square tests were used to compare the proportions of correct identifications made in days two and three versus days four through seven adults, while controlling for gender. The overall association between age and the proportion of insects with correctly identified status was tested with the Cochran-Mantel-Haenszel test. Separately for each sex, the frequency of misidentification types (false diapause versus false non-diapause) was compared between two and seven-day-old adults using chi-square tests. All statistical tests were performed using Sigmaplot 11.0 (Systat Software Inc. 2008).

## Results

The proportion of 10-day-old female bugs that were in diapause (0.69 ± 0.08) was approximately 33% higher than that of male bugs (0.52 ± 0.12; *χ^2^* = 5.356, df = 1, *p* = 0.021), which is consistent with previous reports (Spurgeon and Brent 2010; Brent and Spurgeon 2011). After separating individuals by color, it was found that the occurrence of diapause was 97% for yellow, 56% for light green, and 0% for dark green (Figure 1). The nonzero correlation test of the overall hypothesis of association of color with diapause status was significant (*χ^2^* CMH = 165.1, df = 1, *p* < 0.001). Based on contingency tables controlling for gender, two-sided Fisher exact tests indicated significant associations (*p* < 0.001) between color and diapause status for both females and males.

**Figure 2.**
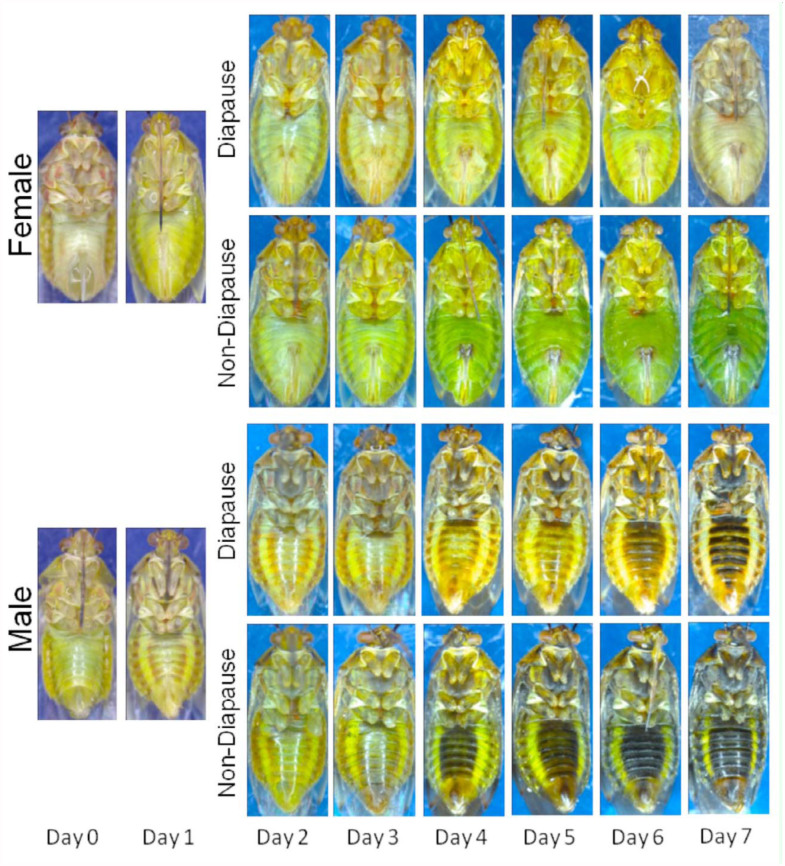
Representative color changes occurring in diapausing and non-diapausing female and male Lygus *hesperus* as they age, starting within a few hours of adult emergence (Day 0) until seven days later. High quality figures are available online.

**Figure 3.**
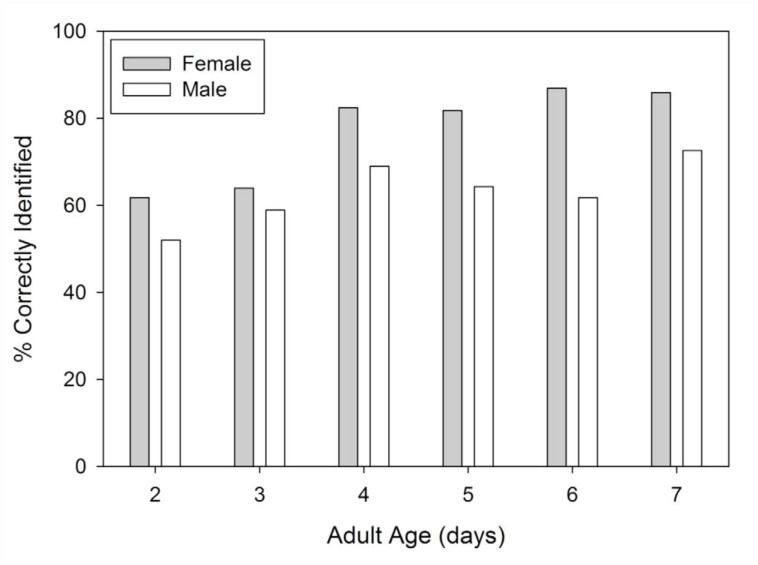
Percent of adult female and male Lygus *hesperus* aged two to seven days for which metabolic status (diapause vs. nondiapause) was correctly identified based on abdominal coloration. For each gender on each day, 25 observers assessed 25 insects. High quality figures are available online.

**Figure 4.**
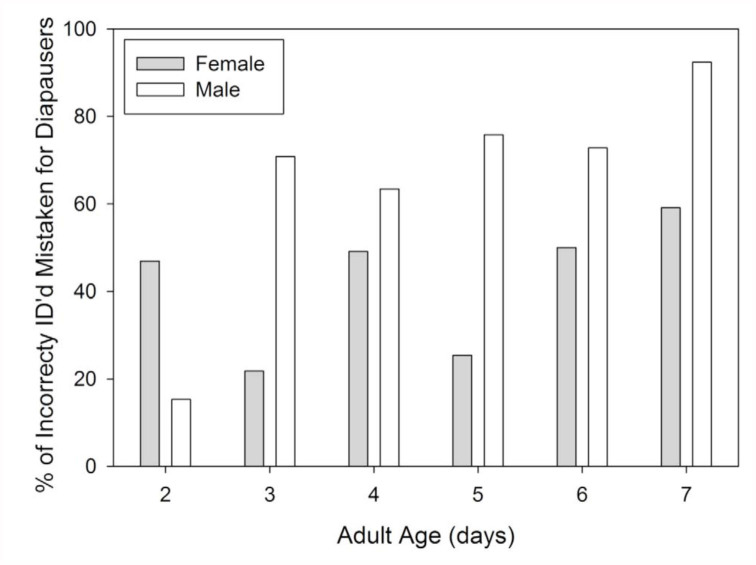
Percent of the adult *Lygus hesperus* whose metabolic status had been incorrectly identified which were mistakenly classified as being in diapause. For each gender on each day, 25 observers assessed 25 insects. High quality figures are available online.

On both the day of and the day following emergence, it was impossible to distinguish between internal development of diapausers and non-diapausers, and the external differences were non-existent. In fact, color differences between diapausers and non-diapausers remained indistinguishable until four days after emergence (Figure 2). The individuals undergoing normal development became increasingly dark in color compared to those in diapause. This continuous color differentiation facilitated the ability of the naïve assessors such that their accuracy increased substantially between days two and seven in both females (+24%) and males (+21%) (Figure 3). The proportion of incorrectly categorized individuals was significantly higher in two to three-day-olds than in four to seven-day-olds for both females (*χ^2^* =207.2, df = 1, *p* < 0.001) and males (*χ^2^* = 41.7, df = 1, *p* < 0.001). Although both genders underwent substantial phenotypic changes as they matured, the status of males and females was not equally discernable. After controlling for age, correct identifications were made at a significantly higher frequency for females (84%) than for males (67%; *χ^2^* CMH = 180.0, df = 1, *p* < 0.001). The types of identification errors also differed between the sexes (Figure 4). While there was no difference in the overall rates with which females were mistakenly categorized as being in diapause or not (*χ^2^* = 2.1, df = 1, *p* = 0.149), the majority of incorrectly identified males were thought to be in diapause (75.5%; *χ^2^* =113.6, df = 1, *p* < 0.001).

**Figure 5.**
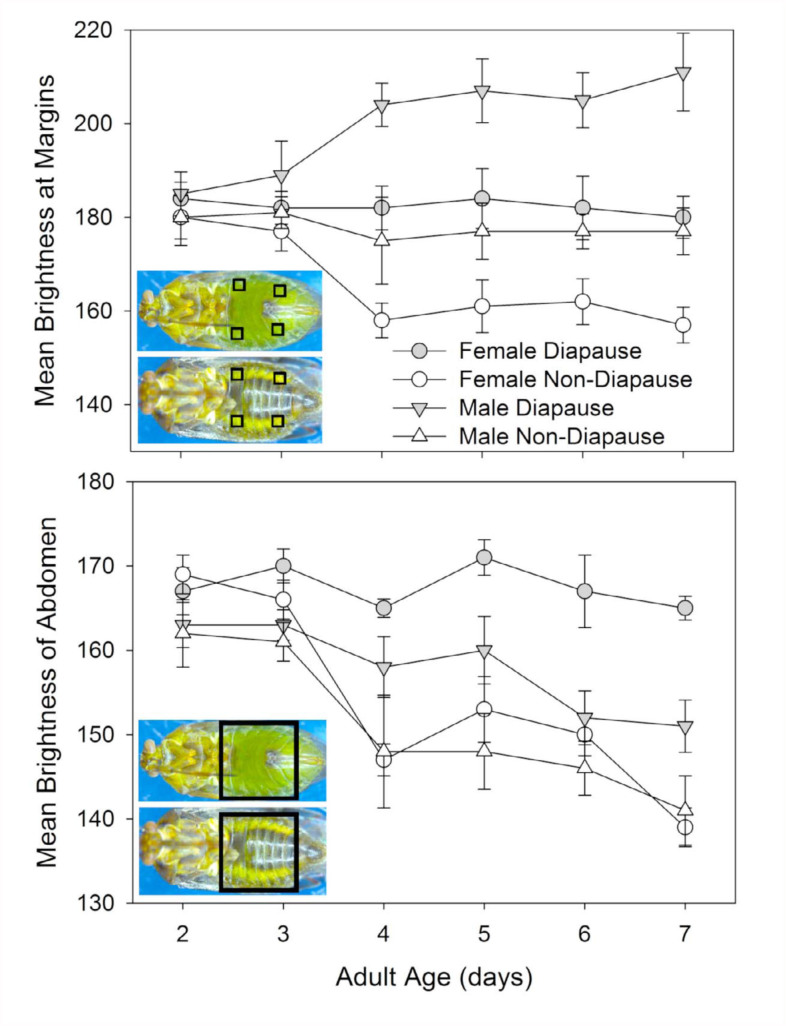
Mean brightness (± SE) scores of diapausers and non-diapausers for both adult female (top picture) and male (bottom picture) *Lygus hesperus* 2–7-days old. Scores were determined based on a sampling of either (A) four 100 × 100 pixel squares in the non-pigmented margins of the sternal cuticle, or (B) a single 1000 × 1000 pixel square covering almost the entire abdomen. Sample size varied from 18 to 25. High quality figures are available online.

The cause for differences in both the number and kind of errors may be in the way that color changed in the males and females. In males, the unpigmented margins of the abdomen (Figure 5a) of non-diapausers retained the same brightness as seen in the youngest adults, but in male diapausers, the brightness increased. This is opposite of the pattern observed with females, in which the brightness of the comparable areas diminished in non-diapausers, but stayed the same in diapausers. The greater brightness of the males compared to the females for these marginal areas would have made color separation more difficult, given that non-diapausing males had a color similar to that of diapausing females. An additional potential impediment to status discrimination among males was their development of substantial black pigmentation on the abdominal cuticle concurrently with the changes to the fat body color (Figure 2). This pigmentation reduced the overall brightness of males as they aged, and may have muted any color contrasts between males in different states to observers with unaided eyes. The analysis for days four through seven that included the pigmented region (Figure 5b) indicated that the overall brightness difference between diapausers and non-diapausers was greater in females (16.4 ± 1.2) than in males (9.7 ± 2.2; t = 2.465, df = 56, *p* = 0.017).

## Discussion

Body color did not appear to be strongly linked to diapause status in *L. hesperus*. In the older individuals examined (Figure 1), coloration was graded from a pale yellow to a dark green, with most diapausers exhibiting the former phenotype, and normally developed individuals exhibiting the latter. This pattern was consistent with previous observations (Beards and Strong 1966; Strong et al. 1970). Individuals with intermediate colors were split between the two states, and may have had a lagging color transformation, or may have been transitioning out of diapause. The latter scenario could come about because the changes in gonadal activity can be quick, particularly for males in which accessory glands fill rapidly (Brent 2010b, c), and may precede changes to body color. Additionally, developmental responses to short photophases can vary even within a cohort of *L. hesperus* reared under identical conditions (Spurgeon and Brent 2010; Brent and Spurgeon 2011), and color response may also exhibit individual variation. Despite these outliers, the linkage between color and diapause status was very strong, at least with 10-day-old individuals who had time to fully develop a phenotype (Brent and Spurgeon 2011). Even in comparatively immature four-day-old individuals, the differences between diapausers and non-diapausers were sufficiently clear to allow accurate categorization (Figure 3).

Age, however, appeared to have had a strong influence on the body color, and may have confounded the use of this metric in the field, where multiple overlapping generations occur. Disruptive linkages to age have been observed in other Heteroptera (Niva and Takeda 2002; Chocorosqui and Panizzi 2003). When developing normally, adult *L. hesperus* of both genders emerge a pale yellow that borders on white. Over the days that follow, their cuticle undergoes rapid sclerotization and more gradual pigmentation (Figure 2). There is evidence that the rate of pigmentation may be slowed in lygus bugs developing under short day conditions (Kelton 1975; Wilborn and Ellington 1984). Adding to the color change is the development of aquacolored adipose tissue covering many surfaces within the hemoceol, and contributing to the fat body (Spurgeon and Brent 2010). In sections of the integument that are semitranslucent, this adipose contributes to the body's green hue. As with the shield bug, *Eurydema oleracea* (Fasulati 1979), diapause appears to interfere with this normal coloration process. In diapausing *L. hesperus*, the hue of the hypertrophied fat body ranges from pale yellow to light green (Spurgeon and Brent 2010), and is only slightly different from the coloration of the externally visible adipose, and the internal organs of very young individuals. Until normal coloration is sufficiently developed, which appeared to be at about four days post-emergence (Figure 2), it was hard to distinguish the diapausing phenotype from an individual that was simply immature (Figure 3).

Gender also impacted the seasonal phenotype of *L. hesperus* such that the diapause status of males was harder to correctly identify based on color (Figure 3). Compared to females, males had a higher proportion of nondiapausing individuals that appeared yellow or light green (Figure 1; 2% vs. 15%), which likely contributed to the over-identification of diapausers (Figure 4). Furthermore, the development of a darkly pigmented swath across the majority of the males' abdomens made it more difficult to discern the color differences. Even though the brightness difference between diapausing and nondiapausing males at the margins of the abdomen were consistently greater than that for females (Figure 5a), the developing black pigmentation caused a decline in the overall brightness of the abdomen, and diminished the apparent divergence between males of different status (Figure 5b). In some cases, this black band could obscure the entire abdomen, making visual categorization impossible. In contrast, diapausing and nondiapausing females, which lacked this black swath, developed increasingly divergent abdomen colors that were readily discerned.

An additional factor which may significantly impact body color is the highly polyphagous nature of *L. hesperus* (Jackson et al. 1995). Although the bugs tested here were provided a uniform diet of green beans, the different plants and parts thereof on which they normally feed in the field will express a wide range of pigments. Plant-derived bilins, carotenoids, and flavenoids can be absorbed and expressed by many insects to produce their distinctive colors (Chapman 1998). Changing the diet of some insects has been shown to produce different color morphs (Van Der Geest 1968; Anazonwu and Johnson 1986; Greene 1989; Yamasaki et al. 2009). This also appears to be true for *L. hesperus*, in which some ingested pigments will readily pass through the gut to become distributed throughout the body (Hoffmann and Hull, unpublished data), and individuals collected from different host plants can have divergent color phenotypes (Wilborn and Ellington 1984). When a lygus bug shifts to a new host plant, its body color may continue to change as new pigments are acquired. These confounding factors point to the impracticably of producing a single color palette for the rapid determination of diapause status. Reliable determination of the relative incidence rate of diapause may require adjustment of the color metric for different crops, although the difference between individuals of different metabolic states reared on the same host plants should still be pronounced.

Collectively, the results suggest that color can be reliably used as an external diagnostic of diapause status when it is not possible to examine internal development. Naïve assessors achieved 75.8% overall accuracy for categorizing adults ≥ 4-days old, and practice can substantially improve this (author's average for the same adults was 90.5%). If the intent is simply to survey for the general incidence of diapause in a population, then further accuracy can be gained by focusing on just the females. If diapausing males need to be pulled out of a mixed population of unknown ages, then it would be best to select only those that have fully pigmented abdomens (and are therefore less likely to be just recently emerged rather than diapausing), and which have very pale abdominal margins. Identifying the impact of environmental factors beyond photophase, such as temperature and diet, on coloration might also enhance predictive accuracy. Similarly, it would be helpful to know whether the cessation of diapause affects internal development and coloration in a coordinated fashion.
